# Nanoarchitectonics: bottom-up creation of functional materials and systems

**DOI:** 10.3762/bjnano.11.36

**Published:** 2020-03-12

**Authors:** Katsuhiko Ariga

**Affiliations:** 1 WPI-MANA, National Institute for Materials Science, 1-1 Namiki, Tsukuba 305-0044, Japan; 2 Department of Advanced Materials Science, Graduate School of Frontier Sciences, The University of Tokyo, 5-1-5 Kashiwanoha, Kashiwa 277-8561, Japan

**Keywords:** bottom-up synthesis, nanoarchitectonics, nanotechnology, self-assembly, supramolecular

**Figure 1:**
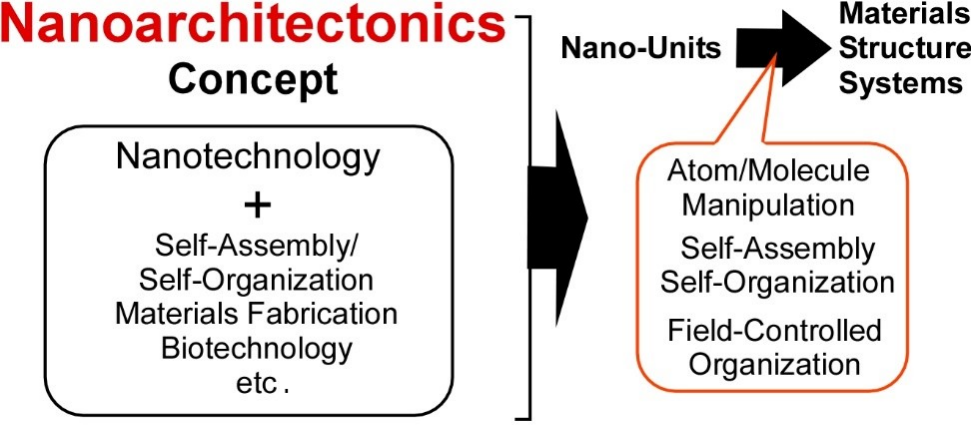
Outline of the nanoarchitectonics concept.

Given the significant and time-critical problems of energy shortage, environmental protection, and biomedical issues, the creation of new functional materials and systems for efficient energy production and storage [1–2], environmental remediation with sensitive pollutant detection [3–4], and biological and biomedical applications [5–6] is a crucial matter. In addition to the intrinsic functionality of bulk materials, control of their internal structure on the nanometer-scale is realized to be increasingly important to obtain high efficiency and specificity in their functions. For this general demand, the bottom-up creation of functional materials and systems from nanometer-scale and molecular units using nanotechnology principles is necessary. This can be accomplished by the conceptual fusion of nanotechnology with the other research fields such as atom/molecular manipulation, organic synthesis, supramolecular chemistry, and bio-related technology. This task is assigned to an emerging concept, nanoarchitectonics (Figure 1) [7–9].

The nanoarchitectonics concept was initially proposed by Masakazu Aono [10–11] who envisioned the production of functional materials with the following principles: (i) construction of functional materials and systems by organizing nanometer-scale structures (nanounits) even with some unavoidable unreliability; (ii) the properties of the structures may differ from those of the individual nanounits, whereby their interactions may synergistically create new functionalities; (iii) unexpected functionality may be included through assembling or organizing a very large number of nanounits; (iv) new theories and computational approaches are developed to support these fabrication processes. Because the features of the nanoarchitectonics concept are general and applicable to most materials systems, this concept can be applied to many research targets. In fact, the nanoarchitectonics concept has already been applied in various fields, including materials production [12–13], structural fabrication [14–15], sensing [16–17], catalysis [18], energy [19], environmental [20], devices [21–22], and bio-related [23–24] applications.

Accordingly, the goal of the thematic issue “Nanoarchitectonics: bottom-up creation of functional materials and systems” was to collect leading research examples that employ the nanoarchitectonics concept. These examples range from fundamental studies on structural formation and control to application-oriented approaches in biology, physical science, and device technology.

As examples of some fundamental studies on the formation and control of nanounits, in one work, the chiral structure was found to control the self-assembly of nitrocinnamic amide amphiphiles [25]. Works related to the formation of higher-dimensional materials included, for example, the self-assembly of crystalline cellulose oligomers that resulted in nanoribbon networks [26], silicon nanowires that were formed by metal-assisted chemical etching (MACE) [27], and the formation of high-tolerance crystalline hydrogels from cyclic dipeptides upon self-assembly [28]. In addition, a review on the use of DNA as the fundamental material building block for molecular and structural engineering [29] gives insight into this interesting field of research which has great potential.

The nanoarchitectonics concept has been applied for various bio-related applications, for example, in the small-protein-induced cellular uptake of complex nanohybrids [30], the controlled drug release from layered double hydroxide/sepiolite hybrids [31], and cell surface engineering with halloysite-doped silica cell imprints for shape recognition of human cells [32]. In another example, magnetic nanoparticles were attached to microbubble shells for enhanced biomedical imaging [33]. In a final example, the detection of the prostate-specific antigen biomarker was expedited by application of advanced data processing and computational tools [34]. The molecular architecture plays a crucial role for obtaining high sensitivity and specificity in immunosensors, thus tools which speed up the ability to analyze the large amounts of data produced could significantly contribute to the field of immunosensing.

Some terrific examples of the application of the nanoarchitectonics concept for engineering applications and the physical sciences include a report by Ruiz-Hitzky et al., where they summarize how photoactive clays incorporating TiO_2_ and ZnO nanoparticles exhibit distinct and useful properties [35]. Other examples include a self-assembled MoS_2_-based composite that was developed for energy conversion and storage purposes [36], a silver-nanoparticle/cellulose-nanofiber composite that was applied for surface-enhanced Raman spectroscopy [37], bio-nanocomposites with clay nanoarchitectures for electrochemical devices [38], a biomimetic nanofluidic diode with polymeric carbon nitride nanotubes [39], and a unique Janus-micromotor applied as a luminescence sensor for sensitive TNT detection [40].

The variety of nanoarchitectonics approaches collected in this thematic issue strikingly demonstrates the wide-range application of this concept. In addition to the bottom-up creation of new functional materials and systems, the inclusion of several additional factors, such as biocompatibility [41] and connection with wet ionic systems [42] that are low cost and emission-less in nature, would facilitate the development for practical usage in the near future.

Katsuhiko Ariga

Tsukuba, February 2020
